# Crossing the Golden Training Divide: The Science and Practice of Training World-Class 800- and 1500-m Runners

**DOI:** 10.1007/s40279-021-01481-2

**Published:** 2021-05-21

**Authors:** Thomas Haugen, Øyvind Sandbakk, Eystein Enoksen, Stephen Seiler, Espen Tønnessen

**Affiliations:** 1 School of Health Sciences, Kristiania University College, Sentrum, PB 1190, 0107 Oslo, Norway; 2 Department of Neuromedicine and Movement Science, Centre for Elite Sports Research, Norwegian University of Science and Technology, 7491 Trondheim, Norway; 3 Department of Physical Performance, Norwegian School of Sport Sciences, 0806 Oslo, Norway; 4 Faculty of Health and Sport Sciences, University of Agder, PB 422, 4604 Kristiansand, Norway

## Abstract

Despite an increasing amount of research devoted to middle-distance training (herein the 800 and 1500 m events), information regarding the training methodologies of world-class runners is limited. Therefore, the objective of this review was to integrate scientific and best practice literature and outline a novel framework for understanding the training and development of elite middle-distance performance. Herein, we describe how well-known training principles and fundamental training characteristics are applied by world-leading middle-distance coaches and athletes to meet the physiological and neuromuscular demands of 800 and 1500 m. Large diversities in physiological profiles and training emerge among middle-distance runners, justifying a categorization into types across a continuum (400–800 m types, 800 m specialists, 800–1500 m types, 1500 m specialists and 1500–5000 m types). Larger running volumes (120–170 vs. 50–120 km·week^−1^ during the preparation period) and higher aerobic/anaerobic training distribution (90/10 vs. 60/40% of the annual running sessions below vs. at or above anaerobic threshold) distinguish 1500- and 800-m runners. Lactate tolerance and lactate production training are regularly included interval sessions by middle-distance runners, particularly among 800-m athletes. In addition, 800-m runners perform more strength, power and plyometric training than 1500-m runners. Although the literature is biased towards men and “long-distance thinking,” this review provides a point of departure for scientists and practitioners to further explore and quantify the training and development of elite 800- and 1500-m running performance and serves as a position statement for outlining current state-of-the-art middle-distance training recommendations.

## Key Points

**Table:** 

This review serves as a position statement for outlining state-of-the-art middle-distance training recommendations.
There are considerable gaps between science and best practice regarding how training principles and training methods should be applied for elite middle-distance running performance.
We identify physiological and training distinctions between world-class 800- and 1500-m runners.

## Background

Middle-distance running was a central part of the Olympic program for men already at the first modern Games in 1896. Over the last century, quantum leaps in men’s performance have been achieved by barrier breaking athletes such as Paavo Nurmi, Gunder Hägg, Rudolf Harbig and Roger Bannister. The progression of female middle-distance running performances was initially slower than that observed for men [1], but this was due to social, not biological constraints. By the 1928 Olympic Games, women competed in 2 of the 13 running events contested by men, the 100 and 800 m. Unfortunately, even this small progress was halted when the International Olympic Committee (IOC) received erroneous reports of female athletes collapsing after running the 800 m and decided to ban women from competing over distances longer than 200 m. The middle-distance events were not added to the Olympic program for women until 1960, after which the sex-gap in middle-distance performance declined gradually until the 1980s. Since then, male and female sex-specific performance differences have stabilized around ~ 10% [2].

Despite an increasing amount of research devoted to middle-distance training [e.g., 3–17], it is reasonable to argue that the developments in these disciplines have not been driven by sport scientists [18]. Publicly available “recipe books” and training diaries based upon the practical experience and intuition of world-leading athletes and coaches have become important and popular sources of best practice training information and framework development for the international middle-distance community [19–59] (Table 1). While best practice training in athletic sprinting [60] and long-distance running [61–65] has been scientifically reported, information regarding the varying training components across the annual cycle of world-class middle-distance runners is limited. Furthermore, the training characteristics of 800- and 1500-m runners have not yet been systematically compared. Such a comparison is warranted because of the marked shift towards a more distinct emphasis on aerobic energy provision from 800 to 1500 m as well as the interactions between mechanical effectiveness and metabolic efficiency in this transition. Therefore, the objective of this review is to integrate scientific and best practice coaching literature to outline a novel framework for the training and development of elite middle-distance performance. Although the present review is anchored in the standard Olympic 800- and 1500-m distances, the outlined terminology, training zone model and training principles are also relevant for other distances and sports.

**Table 1:** Sources of best practice training information

Athletes [reference]	Personal bests (min)	International merits	Type of source
Alberto Juantorena [38]	800 m 1:43.44 (WR)	Olympic gold 1976	Keynote speech/training log
Clayton Murphy [26]	800 m 1:42.93	Olympic bronze 2016	Interview/presentation
David Rudisha [50, 51]	800 m 1:40.91 (WR)	Olympic gold 2012 and 2016	Web post and training log
Hicham El Guerrouj [45]	1500 m 3:26.00 (WR)	Olympic gold 2004	Lectures
Jim Ryun [29]	800 m 1:44.3—1500 m 3:33.1	Olympic silver 1968	Chronicle and training log
Joaquim Cruz [36]	800 m 1:41.77—1500 m 3:34.63	Olympic gold 1984	Chronicle and training log
John Walker [28]	1500 m 3:32.4—mile 3:49.08 (WR)	Olympic gold 1500 m 1976	Magazine article/interview
Marty Liquori [39]	Mile 3:52.2	Pan American champion 1971	Chronicle and training log
Michael Rimmer [40]	800 m 1:43.89	EC silver 2010	Chronicle and training log
Natalia Rodriguez [43]	1500 m 3:59.51	WC and EC gold 2010–2011	Chronicle
Nick Symmonds [30]	800 m 1:42.95—1500 m 3:34.55	WC silver 2013	Training log
Nick Willis [44]	1500 m 3:29.66—mile 3:49.83	Olympic medals 2008 and 2016	Training log
Peter Elliott [22]	800 m 1:42.97—1500 m 3:32.69	Olympic silver 1988	Chronicle and training log
Said Aouita [24]	1500 m 3:29.46 (WR)—mile 3:46.76	Olympic gold 1984, WC gold 1987	Training log
Silas Kiplagat [49]	1500 m 3:27.64	WC silver 2011	Training log
Taoufik Makloufi [46]	800 m 1:42.61—1500 m 3:28.75	Olympic gold 2012	Interview

Coaches [reference]	Successful middle-distance athletes	Athlete merits	Type of source
Arthur Lydiard [19–21]	Peter Snell (WR), Murray Halberg, Barry Magee	Olympic gold 1960 and 1964	Books
Bill Bowerman [53]	Steve Prefontaine, Jack Hutchins, Sig Ohlemann	He trained 31 Olympic athletes	Book
David Sunderland [52]	Jane Finch, Lynsey Sharp	Indoor WR 1977, EC gold 2012	Book
Gianni Ghidini [37]	Wilfred Bungei, Amel Tuka	Olympic & WC medals since 2001	Presentation
Harry Wilson [34, 57]	Steve Ovett (WR)	Olympic gold 1980, EC gold 1978	Chronicle/training log
Honore Hoedt [41]	Sifan Hassan (WR)^a^, Brad Som, Amoud Okken	WC & EC medals since 2006	Presentation
Jack Daniels [58]	Coached seven athletes to the U.S. Olympic team	Olympic finalists	Book
Jama Aden [31]	Genzebe Dibaba (WR), Abdi Bile, Taoufik Makloufi	Olympic & WC medals since 1987	Magazine article/interview
Joe Vigil [33, 56]	Coach for the US Olympic team in 1998	Olympic finalists	Presentations
Kim McDonald [23]	Daniel Komen (WR), Noah Ngeny, Laban Rotich	Multiple WC medals in the 1990s	Chronicles/training logs
Lee LaBadie [26]	Clayton Murphy	Olympic bronze 2016	Presentations
Margo Jennings [32]	Maria Mutola, Kelly Holmes	Olympic & WC medals 1993–2004	Chronicle/interview
Nic Bideau [48]	Craig Mottram	WC bronze 2005	Commentary
Peter Coe [54, 59]	Sebastian Coe (WR)	Olympic gold 1980 and 1984	Books
Steve Magness [42]	Assistant coach and scientific advisor for elite runners	Olympic & WC medals 2011–2012	E-book and presentation
Tomasz Lewandowski [25]	Marcin Lewandowski	EC gold 2010, WC bronze 2019	Presentation
Vin Lananna [35]	U.S. Olympic team coach	Olympic finalists	Presentations

In addition, we have had personal communications with Vebjørn Rodal (Olympic 800-m champion in 1996) and Arturo Casado (European 1500-m champion in 2010). Novel training data from these athletes are presented in Table 6

*WC *world championships, *EC *European championships, *WR *former or current world-record holder

^a^Honore Hoedt coached Sifan Hassan during her early career, not when she broke several world records

The present review strategy is challenging. Firstly, an initial review of the literature reveals that several biases are present, including a substantial sex bias (male dominance) as well as “group culture” biases across a handful of successful training groups. A relative bias towards emphasis on training aerobic capacity is particularly present for the 800 m, as this discipline seems heavily influenced by “long-distance thinking” in the available research literature. Hence, the generalizable training recommendations outlined in this review might not be optimal for all middle-distance athletes. Secondly, a potential source of misinterpretation is the lack of a common framework and terminology. Moreover, the included coaching literature cannot be controlled for possible training prescription-execution differences as exemplified by Ingham et al. [9]. Although these stories rarely gain attention, most “famous” coaches have also coached underperforming talents. We acknowledge this bias but note that the vast majority of the coaches listed in Table 1 have achieved success with multiple athletes. Finally, the widespread use of doping in international athletics must be acknowledged. All these challenges and limitations reflect today’s athletics, for better and worse, and the outcomes of this review must therefore be interpreted with these caveats in mind. Sensitive to these limitations, we still contend that integration of available research evidence and results-proven practice provides a valid point of departure for outlining state-of-the-art training recommendations and for generation of new hypotheses to be tested in future research [60, 66].

## Physiological and Mechanical Determinants of Middle-Distance Running Performance

The 800- and 1500-m running disciplines are where aerobic and anaerobic energetics converge [5]. Importantly, these classically defined disciplines are also where effective maximal sprint speed (MSS) mechanics and efficient long-distance running energetics collide. While mechanics and energetics are not independent in middle-distance running, we choose to examine these events with what might be called scientific bifocals and try to converge them in a logical manner.

### The Energetic Side of the Middle-Distance Coin

During an 800-m run, the relative energy system contributions from aerobic and anaerobic metabolism are reported to be 60–75 and 25–40%, respectively, while corresponding values for 1500 m are 75–85 and 15–25% [6, 7, 13]. The range in energy system contribution is greater in the 800 m compared to the 1500-m event due to the variability of the athletes presenting at 800 m. Overall, these relative aerobic energy contribution estimates overlap reasonably well with the reported type I muscle fiber distribution ranges in middle-distance runners [13]. Just as has been well established for long-distance running, maximal oxygen uptake (*V*O_2max_), fractional utilization of *V*O_2max_, running economy (RE), velocity at the anaerobic threshold (vAT), and velocity at *V*O_2max_ (*vV*O_2max_) are all positively correlated with middle-distance performance [5, 8, 67]. However, to optimize energy mobilization and utilization, O_2_ kinetics as well as anaerobic power and capacity play decisive roles in middle distance performance. As Olympic gold medalist 800-m runner Vebjørn Rodal succinctly summarized the importance of O_2_ kinetics to one of the authors (ØS): “It does not matter if I can reach a higher *V*O_2max_ in five minutes when I have to cross the finish line in 102 s.” In addition, both energy expenditure capacity and economy/efficiency likely deteriorate during middle-distance events, indicating that fatigue-resistance/resilience might have a decisive performance-impact. To this point, de Koning and colleagues have directly challenged the assumption of a stable gross efficiency during short maximal cycling efforts within the middle-distance time window [68, 69]. Using a sequence of sub-maximal-maximal-sub-maximal trials and back-extrapolation, they estimate that metabolic efficiency declines enough during 100–240 s duration cycling time trials to result in a ~ 30% underestimation of the anaerobic energy contribution to total energy expenditure. Unfortunately, comprehensive quantification of running economy (total external work performed/total energy expenditure) at speeds above the lactate threshold remains elusive [12].

While traditional endurance disciplines can be described as maximization challenges (i.e., training that enhances *V*O_2max_ or fractional utilization is “always positive” for performance), we propose that the 800-m event in particular requires an energy release optimization strategy that respects the interactions and trade-offs between anaerobic and aerobic metabolism emerging in both training and performance. This complexity allows internationally successful middle-distance runners to present a variety of physiological profiles [12–15]. For example, *V*O_2max_ ranges from ~ 65 to 85 ml·kg·min^−1^ in elite men [16, 29, 70, 71]. Similar variation is seen among elite women, albeit at ~ 10% lower values [71] due to lower hemoglobin concentrations and higher relative body fat percentage [72]. Consequently, correlations between isolated aerobic performance-determining factors and performance in homogeneous subsets of middle-distance runners are modest at best.

We find no evidence to suggest that female and male middle distance athletes should not be examined as one elite population from an energetics point of view. However, the 800-m event rides an energetic “tipping point;” it sits on a portion of the velocity-duration curve where the aerobic and anaerobic contributions are particularly duration sensitive. Consequently, the additional ~ 15 s required to complete the 800 m by the best females may nudge this event towards the aerobic end of the training spectrum enough that it alters the optimal composition of their training compared to male counterparts. Lending some support to this possibility, we note that inspections of the top 200 all-time lists for the 800 and 1500 m reveal that 55 women appear on both lists, compared to only 38 men (http://worldathletics.org). For comparison, the 1500–3000 m double is more common among the 200 all-time best males and females with 51 men and 78 women appearing on both lists.

### Mechanical Effectiveness: The Other Side of the Middle-Distance Coin

The role of anaerobic capacity in middle-distance running has received considerably less attention in the research literature, likely due to limitations in accurately and reliably quantifying anaerobic energetics [73]. Bachero-Mena et al. [3] have reported a strong relationship between 800-m performance and sprints over 20 m (*r* = 0.72) and 200 m (*r* = 0.84) in male national and international 800-m runners (1:43–1:58). Peter Coe [54] and Arthur Lydiard [19] have argued that world-class 800-m male athletes should be able to run 200 m in < 22.5 s prior to major competitions. Such sprint performance is determined by a combination of anaerobic energy release and the ability to transfer energy to speed over this particular distance, and this sprinting capacity requirement eliminates at least 99% of males on the planet as future world-class 800-m runners before other physiological demands are even considered. Power output and technique are considered key underlying determinants for MSS [74]. Fast male world-class middle-distance runners may approach 10 m·s^−1^ [12, 15], and if we assume a ~ 10% sex difference [75], corresponding females are capable of sprinting ≥ 9 m·s^−1^. To achieve such running velocities, maximal horizontal power outputs of ~ 21 and ~ 19 W·kg^−1^ are required for men and women, respectively [76].

Although the basic principles of MSS are relatively simple and governed by the laws of motion, the way an athlete solves the mechanical constraints and utilizes the degrees of freedom within these constraints is far more complex [74]. Spatiotemporal variables, segment configuration at touchdown and lift-off, lower-limb segment velocities immediately prior to touchdown or during ground contact, leg stiffness, storage and release of elastic energy, as well as front- and back-side mechanics have received much attention in research literature. However, these mechanical variables are entangled, and no single variable is associated with better MSS [74]. For more information regarding running mechanics, we refer to previously published biomechanical analyses [e.g., 74, 77, 78].

Overall, middle-distance athletes must be able to reach high MSS if they are to reach an international level. However, high and unfatigued MSS is not useful if a high percentage of that velocity cannot be maintained for 100–240 s (see Sect. 3). This implies a complex integration of muscular power, metabolic efficiency, biomechanical efficiency and fatigue resistance at the muscle fiber level, as well as an optimal pacing strategy [79, 80].

## Athlete Profiling

Due to the variety of physiological profiles among 800- and 1500-m runners, coaches typically categorize middle-distance runners into distinct “types” [19–21, 41, 47, 54, 58, 59], and these types bear different labels (e.g., “speed-based” vs. “endurance-based”, “fast-typed” vs. “stamina-typed”). A simple method for athlete profiling and identification of individual strengths and weaknesses can be based on performance across a spread of distances below and above the main discipline (e.g., using IAAF points or percent time behind current world record). For example, 400, 800 and 1500-m performance can form the basis for analyzing an 800-m runner, presupposing that the performance level across all these distances is representative and reflects actual performance [13]. A brief review of the World Athletics all-time top lists (https://www.worldathletics.org/records/all-time-toplists) clearly shows that 1500-m runners possess a broader distance performance range, while a larger proportion of world-class 800-m runners appears to be “specialists”. These observations are in accordance with Daniels [58], who argued that a strong performance relationship exists among distances ranging from 1500 m to marathon in heterogeneous subsets, while 800 and 1500 m performances are considerably less related.

The concept of anaerobic speed reserve (ASR) was originally introduced by Blondel et al. [81] and further developed by Sandford and associates [12–15] to provide a “first layer insight” of athlete profiling. ASR is defined as the speed zone ranging from *vV*O_2max_ to MSS. MSS can be accurately measured using radar technology or timing gates [82, 83], while *vV*O_2max_ (also known as maximal aerobic speed; MAS) traditionally has required laboratory-based procedures. However, a field method has recently been developed where a regression equation can be applied for accurate prediction of *vV*O_2max_ from 1500 m time-trial performance (“gun-to-tape” or “predicted 1500-m shape”) [14]. Based on the speed reserve ratio concept (SRR = MSS/MAS), Sandford and associates classified 800-m runners into three sub-groups along a continuum as follows: 400–800 m types (SRR ≥ 1.58), 800 m specialists (SRR ≤ 1.57 to ≥ 1.47, and 800–1500 m types (SRR ≤ 1.47 to ≥ 1.36) [15]. Using the same approach, we propose that 1500-m runners can be categorized as 800–1500 m types, 1500-m specialists and 1500–5000 m types. However, the validity of this concept must be further elaborated in future research. In the following sections of this review, the implications of athlete profile for training prescriptions will be explored in more detail, with most focus on the distinctions between 800- and 1500-m runners.

## Expected Performance Development Among Elite Middle-Distance Runners

Middle-distance performance capacity evolves and devolves throughout life via growth, maturation, training and ageing [84–87]. The age of peak performance in world-class middle-distance runners (mean ± SD) is 25–27 ± 2–3 years [87–90]. However, training age must also be considered, as early/late specialization may accelerate/delay age of peak performance [91]. For example, young African runners have a lifestyle that includes running to and from school from a very early age [23, 27, 92, 93], supporting the early engagement hypothesis [94]. However, history has also shown that late specialization and diversified experience in other sports can provide a platform for later elite performance [17, 36, 38, 39].

For the very best runners, the annual within-athlete performance differences are lower than the typical variation and the smallest worthwhile change is ~ 0.5% in middle-distance running [95]. Mean annual improvement scores for the world’s top 100 middle-distance runners in their early twenties are in the range of only 0.1–0.2% [87]. On average, athletes must be at a very high level already in their late teens to become world-class as seniors. Haugen and co-workers calculated that middle-distance runners within the annual world top 100 lists averaged 98–99% of their peak performance result at the age of 20 [87]. However, athletes reaching the upper portion of this exclusive annual list improve their performances more than athletes of lower performance standards in the years immediately preceding peak performance age [87]. These differences may be explained by differences in training status, responsiveness to training, coaching quality, doping, etc. Although there is considerable variation among athletes and numerous routes to expertise under optimal conditions, a review of the best practice literature listed in Table 1 indicates that the majority of world-class 800- and 1500-m runners have specialized in the middle-distances already as juniors.

## Training Principles

### Progressive Overload

The process of training adaptation is an interplay between loading and recovery, and the principle of progressive overload refers to the gradual increase of stress placed upon the body during exercise training [96–98]. Indeed, the capacity to perform and absorb large training loads is seen as both an adaptation over time and a talent. In middle-distance running, commonly reported external load factors include volume, duration and intensity, while psychophysiological internal load factors typically include heart rate, blood lactate and session rating of perceived exertion. These variables will be examined in more detail in Sect. 6. While running distance is the most commonly reported loading factor in scientific and best practice literature, some authors argue that rating of perceived exertion (RPE) or training impulse (TRIMP; min × RPE) are more useful for the training decision-making process [99, 100]. With emerging and novel wearable technology, future training monitoring may put more emphasis on biomechanical external load metrics such as tibial shock, foot-strike angle, ground contact time and leg stiffness to enable a more precise quantification of training stress [99].

The principle of progressive overload is envisioned to enhance performance over time and reduce the risk of injury and overtraining [96–98]. Indeed, a large proportion of injuries are attributed to rapid and excessive increases in training load [101, 102]. During the initial 8–12 weeks of the training year, it is therefore widely accepted in the middle-distance community that running volume must be increased gradually. In elite athletes, the initial training week is performed with ~ 40–60% of peak weekly running volume, increasing by ~ 5–15 km each week until maximal volume is reached [19–26, 28–32, 34, 36–46, 52, 54–59]. This increase is mainly achieved by increasing training frequency in the initial phase, then subsequently extended by lengthening individual training sessions. When peak running volume is achieved, the further progression in training load among middle-distance runners is normally achieved by increasing the amount or intensity of intensive training. Long-term progression rates depend on training experience and individual predispositions, but total training volume and peak weekly mileage may increase up to ~ 10% per year during the late teens in well-trained athletes [17, 42, 55, 56].

A common “periodization” approach observed within best practice is that more intensive training sessions are introduced and total training volume decreases as the competition season approaches [17, 19–21, 23–25, 34, 36, 40–42, 50–52, 54–56, 58, 59] (see also Sect. 5.4). Within this context, running surface and footwear are crucial modifiers of training load for middle-distance running. It is generally assumed that the harder the surface, the higher mechanical load and reactive forces on lower limb tissues [19–21, 23, 36, 52, 54–59, 99]. Most elite athletes perform low-intensive running sessions with cushioned running shoes/trainers on forgiving surfaces (forest trails, parkland, dirt road, etc.), while high-intensive running and sprinting sessions are performed with spike shoes on a rubberized track surface. Because the latter is associated with high muscular load, such sessions rarely occur on consecutive days among leading coaches and practitioners [17, 19–21, 23–25, 31, 34, 36, 40, 41, 50–52, 54–56, 58–60].

Although altitude training is an integrated part of modern middle-distance training to increase the stress placed upon the body, this topic has received limited attention in the best practice coaching literature. We therefore refer to previously published reviews for more information regarding altitude training [e.g., 103–105].

### Specificity

Training adaptations are specific to the stimulus applied, encompassing muscle groups and actions involved, speed of movement, range of motion and energy systems involved [98, 106]. Due to the performance demands underpinning middle-distance running performance, various types of training aimed to overload the aerobic and/or the anaerobic energy system while employing movement patterns specific to middle-distance running need to be performed. Based on a synthesis of best practice literature [19–59], the specific training methods for middle-distance running are described in Table 2. We refer to previously published review papers regarding physiological adaptations and responses associated with such training forms [6, 7, 107–109].

**Table 2:** Specific training methods for middle-distance running

	Training method	Description
Continuous running	Warm up/recovery run/cool down	Low-intensive running (typically 3–5 km·h^−1^ slower than marathon pace, i.e., 4:00–4:45 and 4:30–5:15 min·km^−1^ for men and women, however, the last part of the warm-up may approach marathon pace or slightly above), predominantly performed on soft surface (grass, woodland, forest paths, etc.). Typical duration is 10–30 min
	Long run	Low-intensive steady-state running (marathon pace or 1–2 km·h^−1^ slower, i.e., 3:30–4:00 and 4:00–4:30 min·km^−1^ for men and women) performed on forgiving surfaces such as forest trails where possible. Typical duration is 60–90 min, but 2-h runs are also performed during the preparation period
	Anaerobic threshold run	A sustained run at moderate intensity/half-marathon pace (i.e., 2:55–3:15 and 3:10–3:30 min·km^−1^ for world-class male and female middle-distance runners). Typical duration 15–40 min. The session should not be extremely fatiguing
	Fartlek	An unstructured long-distance run in various terrains over 30–60 min. where periods of fast running are intermixed with periods of slower running. The pacing variations are determined by the athlete’s feelings and rhythms and terrain
	Progressive long runs	A commonly used training form used by African runners. The first part of the session is identical to an easy long run. After about half the distance, the pace gradually quickens. In the final portion, the pace increases to the anaerobic threshold (half-marathon pace) or slightly past it. Athletes are advised to slow down when the pace becomes too strenuous
Interval training	Anaerobic threshold intervals	Intervals of 3–10 min. duration at an intensity around anaerobic threshold (half-marathon pace) or slightly faster. Typical sessions: 8–12 × 800–1000-m with 1 min. recovery between intervals, 4–8 × 1500–2000 m with 1–2 min. recovery between intervals, or 2–4 × 10-min. with 2–3 min. recovery between intervals. As a rule of thumb, the recovery periods are ~ 1 min. of easy jogging per 5 min of running. Recommended total time for elite runners is 25–40 min. Such intervals are advantageous because they allow the athlete to accumulate more total time than during a continuous anaerobic threshold run
	*V*O_2max_ intervals	Intervals of 2–4 min. duration at 3–10 K pace, with 2–3 min. recovery periods between intervals. Typical sessions: 4–7 × 800–1000 m or 2 × (6 × 400 m) with 30–60 s and 2–3 min. recovery between intervals and sets, respectively. Recommended total time for elite runners is ~ 15–20 min
	Lactate tolerance training	Intervals typically ranging from 200 to 600 m with 800–1500 m race pace and 1–3 min. recoveries. Typical sessions: 10–16 × 200 m with 1 min. recovery between intervals, or 3 x (4 × 400 m) with 60–90 s and 3–5 min. recoveries between intervals and sets, respectively. Total accumulated distance ranges from 1500 to 5000 m in elite athletes
	Lactate production training	Intervals typically ranging from 150 to 600 m at 200–600 m race pace and full recoveries. Typical sessions: 5–7 × 300 m with 3–5 min. recoveries, 3–5 × 400 m with 7–15 min. recoveries, or 600–500–400–300–200 m with 6–15 min. recoveries. Total accumulated distance ranges from 800 to 2500 m in elite athletes
	Hill repeats	The main intention is overloading horizontal propulsive muscle groups while reducing ballistic loading. Typical incline is 5–10%, and duration vary from ~ 15 s to ~ 4 min. depending on intensity, goal (aerobic intervals, lactate production or tolerance training) and time of season. Typical sessions: 10–15 × 100 m with 60–90 s recoveries, or 6–8 × 800–1000 m with easy jog back recoveries. Hill repeats are mainly performed during the preparation period
Sprints or time trials	Time trials	“All-out” efforts or trials aiming at achieving a target time. Distances are normally 50–80% of the athlete’s normal racing distance. Typically performed prior to (e.g., 10 days) an important race at the early part of the season
	Sprints	5–15 s runs with near-maximal to maximal effort and full recoveries. These can also be performed as strides, progressive runs or flying sprints, where the rate of acceleration is reduced to allow more total distance at higher velocities. The main aim of the session is to develop or maintain maximal sprinting speed without producing high levels of lactate

Many successful athletes in typical endurance sports supplement their sport-specific training with alternative activity forms, so called cross-training [110–113]. Arguments supporting the inclusion of such non-specific training include injury prevention, aerobic capacity benefits, strengthening “weak links”, and avoidance of training monotony [113, 114]. Best practice coaching literature within middle-distance running indicates that cross-training (e.g., cycling, swimming, running with floating vest or cross-country skiing) in most cases is employed during injury rehabilitation processes. However, it cannot be precluded that this is a part of the regular plan in certain training groups. Other “less specific” training forms such as strength, power and plyometric training are more commonly performed to target the underlying anaerobic performance components (see Sect. 6.4). Although these training forms do not duplicate the holistic running movement, they may target specific components that limit performance.

### Individualization

The majority of training intervention studies demonstrate that considerable variability in adaptation to a given exercise stimulus is the norm [e.g., 115–117]. The principle of individualization refers to the notion that training prescription must be adapted and optimized according to individual predispositions (performance level, training status/age, sex, recovery/injury status and physiological and structural/mechanical profiles) to maximize the effect and avoid non-responder outcomes [13, 52, 58, 98, 118]. Total training load is typically higher in well-trained adult runners of higher performance standard compared to their younger, less trained and lower-performing counterparts [19–21, 56, 58]. A review of the best practice literature reveals that world-class middle-distance athletes have recorded very similar personal best times with substantial differences in training programs, and these differences are likely related to the varying physiological and profiles that exist within and between 800- and 1500-m runners (see Sect. 6).

### Variation and Periodization

The principle of variation refers to the concept that systematic variation in training is most effective for eliciting long-term adaptations [98, 119]. The most commonly investigated training theory involving planned training variation is periodization, an often-misused term that today refers to any form of training plan, regardless of structure [119]. Ever since Arthur Lydiard introduced his periodization system in the late 1950s [19–21], leading practitioners within middle-distance running typically divide the training year (macrocycle) into distinct, ordered phases to peak for important competitions [23–26, 28, 31, 32, 34, 36–38, 40, 42, 43, 45, 52, 54–57, 59]. At least three phases are typically organized within a macrocycle: a preparation period, a competition period and a transition period. The transition period begins immediately after the outdoor competition season, typically consisting of 2–4 weeks with rest or recreational training. The following preparation period is typically broken up into *general* and *specific* preparation. Some athletes apply double periodization (i.e., two peaking phases), consisting of a preparation phase, an indoor season, a new preparation phase and finally an outdoor competition season [24, 32, 43]. However, most world-class middle-distance runners apply single periodization. Although they may participate in cross-country or indoor competitions during their preparation phase, such competitions mainly serve as a refreshing change from daily training.

The historical development underlying today’s practices for variation and periodization among world-class middle-distance runners is described in Table 3. The training organization models outlined in the 1950s, 1960s and 1970s are still valid, as we and others have systematically quantified the training of successful endurance athletes in a range of sports and reported a “polarized” (i.e., significant proportions of both high- and low-intensity training and a smaller proportion of threshold training) [122, 123] or pyramidal (i.e., most training is at low intensity, with gradually decreasing proportions of threshold and high-intensity training) intensity distribution [124]. Modern endurance training practice among elite performers in numerous sports [110–112, 125–132] is dominated by frequent sessions and high total volumes of low intensity training combined with smaller volumes of high intensity training organized as 2–4 “key workouts” in most training weeks. This training organization also holds true for well-trained and world-leading middle-distance runners [10, 16, 17, 22–59, 133], although 800-m runners apply a greater proportion of training at higher intensities than 1500-m runners (see Sect. 6.3). We argue that the ubiquitous nature of this basic intensity distribution across sports with very distinct “cultures and training histories” suggests some physiologically rooted self-organizing forces at play related to sustainably balancing cellular signaling and systemic stress over time. However, the long-term and cross-disciplinary influence of groundbreaking coaches cannot be discounted.

**Table 3:** An historical overview of middle-distance training organization

New paradigms	Key coaches and athletes driving the development
**1920s** Use of systematic methodologies targeting middle-distance running	Paavo Nurmi was the pioneer of interval training and introduced the “even pace” strategy to running, using a stopwatch to control his speed [120]. He also developed systematic all-year-round training programs that included both long-distance work and high-intensive running [1], bringing middle- and long-distance training to a new and modern level with intelligent application of effort
**1930s** Introduction of interval concepts and use of heart rate for intensity control	German Waldemar Gerschler (coach of e.g. Harbig and Moens) together with the physiologist Herbert Reindell refined the interval training concept [1]. The intensity in each interval was carefully controlled by heart rate and typically higher than competition pace interspersed by short breaks
**1940s** Introduction of “fartlek” as a training method	Swedish Gösta Holmer (coach of e.g. Hägg and Anderson) developed “fartlek” as a training method [1], an unstructured long-distance run in various terrains where periods of fast running are intermixed with periods of slower running
**1950s** Use of high-volume low intensity running as a basis of middle-distance running Gradually reduced volume and more competition-specific speed/intensity towards the competition period	New Zealander Arthur Lydiard (coach of e.g. Snell and Halberg) broke with contemporary practice by prescribing a large volume of low intensity running to his middle-distance athletes, peppered with specific high-intensity training, hill bounding and plyometric training [19–21] The emphasis on high-volume aerobic training shifted towards less volume and more specific anaerobic and race-specific workouts towards the competitive season, which remains the foundation for most modern training programs. This training model bears great resemblance to Matveyev’s traditional training periodization [121]
**1960s** Systematic micro-periodization of hard and easy workouts	Oregon and USA track and field coach Bill Bowerman popularized the hard/easy principle of running; days of hard workouts (e.g., interval training) were systematically alternated with easy days of low-intensive running [53]
**1970–1980s** Introduction of the multi-pace training concept Use of 2–3-day clustering of anaerobic sessions	In the 1970s, Frank Horwill, the founder of the British Milers’ Club, formulated and innovated the multi-pace training concept [47]. This system involves training at four or five different combinations of paces and distances in a 10–14-day cycle. The distances are rotated so that over-distance, event-specific and under-distance paces are all covered. Horwill’s training philosophy deviates from Lydiard's, both in terms of ~ 50% less weekly running volume, as well as larger amounts of anaerobic training throughout most of the macrocycle. This system has been utilized by several world-leading middle-distance athletes, including Sebastian Coe [54, 59], Said Aouita [24], Hicham El Guerrouj [45], Maria Mutola and Kelly Holmes [32] Another characteristic feature that emerged in British middle-distance running in the 1970s and 1980s was the 2–3-day clustering of anaerobic sessions (high-intensive intervals, strength, power and plyometric training), followed by 1–2 low-intensive (aerobic) training days [47, 54, 57, 59]. This micro-periodization model involves an alternate taxing of the cardiovascular and neuromuscular systems, also described as a reduced form of “crash training”. This philosophy has later been used by several world-leading middle-distance athletes [14, 37] (Table 6)
**2000–2010s** Introduction of the polarized and pyramidical intensity distribution concepts	Several acknowledged scientists systematically quantified the training of successful endurance athletes in a range of sports and reported a “polarized” (i.e., significant proportions of both high- and low-intensity training and a smaller proportion of threshold training) [110, 111] or pyramidal (i.e., most training is at low intensity, with gradually decreasing proportions of threshold and high-intensity training) intensity distribution [112]. Accordingly, this training organization holds true for most of today’s world-leading middle-distance runners

## Training Characteristics

### Training Quantification Considerations

While training volume in typical endurance sports can be quantified in a straightforward manner using number of sessions, hours and kilometers, quantification of training intensity is more complicated. In scientific studies of elite endurance athletes, 3- or 5-zone intensity scales have been developed based on either external work rates (running pace or types of training), internal physiological responses (*V*O_2_, blood lactate and/or heart rate ranges) or how the training was perceived [62, 110–112, 125–129]. These previously developed scales are not applicable for middle-distance runners because (1) parts of their training are performed at considerably higher intensities, and (2) middle-distance athletes exhibit physiological training responses different from aerobic endurance athletes (e.g., higher blood lactate levels). Acknowledged and leading middle-distance practitioners have developed alternative training zone models [17, 54, 56, 58, 59], but no consensus has been established. However, describing and comparing training characteristics requires a common intensity scale. To identify the training differences between 800- and 1500-m runners in more detail, we have developed a 5- and 9-zone intensity model (Table 4) based on an integration of scientific [17, 62, 110–112, 122–129, 134] and best practice coaching literature [54, 56, 58, 59].

**Table 4:** Intensity scale for elite middle-distance runners

Scale		BLa	HR	*V*O_2max_	RPE	TTF	Race pace	AWD	Int. time	Rec	Training methods
9-zone	5-zone	mmol·L^−1^	% max	%	6–20	min		min·session^−1^	min	min	
9	SST	n/a	n/a	n/a	n/a	< 0:08	≤ 60 m	< 1	< 0:08	1–3	Accelerations, flying sprints (alactic)
8	SST	n/a	n/a	n/a	n/a	0:15	60–120 m	1–3	< 0:15	1–3	Progressive runs or maximal sprints
7	VHIT	> 12	n/a	115–140	19–20	1	120–600 m	3–6	0:15–1:30	3–15	Lact. prod. training, TT, LS competitions, hill rep
6	VHIT	> 12	n/a	100–114	19–20	4	800–1500 m	6–15	0:25–1:30	0:30–3	Lact. tol. training, TT, MD competitions
5	HIT	8.0–12.0	> 93	90–99	18–20	15	3000–5000 m	15–25	1–4	1–3	*V*O_2max_ int., LD competitions, hill rep
4	HIT	4.0–8.0	88–92	85–89	16–18	30	10 000 m	20–35	2–7	1–3	*V*O_2max_ int., hill rep
3	MIT	2.5–4.0	83–87	80–84	14–16	60	Half-marathon	20–50	3–10	1–2	AT runs, fartlek, AT int., prog. runs^b^
2	LIT	1.5–2.5	73–82	70–79	12–14	120	Marathon	20–90	n/a	n/a	Long run
1	LIT	< 1.5	60–72	55–69	9–12	n/a	n/a	20–150^a^	n/a	n/a	Recovery run, easy long run

*BLa* typical blood lactate (normative blood lactate concentration values based on red-cell lysed blood), *HR* typical heart rate, *VO*_*2max*_ maximal oxygen consumption, *RPE* rating of perceived exertion, *TTF* time to fatigue (single effort), *AWD* typical accumulated work duration, *Int*. interval, *Rec*. typical recovery time (active or passive) between repetitions, *prog*. progressive, *lact*. *prod*. lactate production, *lact*. *tol* lactate tolerance, *hill* *rep*. hill repeats, *AT* anaerobic threshold, *TT* time trials, *LS* long-sprint, *MD* middle-distance, *LD* long-distance, *LIT* low-intensity training, *MIT* moderate intensity training, *HIT* high-intensity training, *VHIT* very high-intensity training, *SST* short-sprint training

^a^Warm-up is typically performed in zone 1–3, although with shorter duration, while cool downs are typically performed in zone 1–2

^b^Progressive runs are typically performed in zone 1–3

Standardized intensity scales can be criticized for several reasons. Firstly, they fail to account for individual variation in the relationship among physiological variables (e.g., between heart rate and blood lactate concentration) [123]. Secondly, the method of training intensity quantification can affect the computation of the training intensity distribution [135]. Thirdly, prescribing exercise intensity based on a fixed percentage of maximal physiological anchors (e.g., *V*O_2max_ or maximal heart rate) has little merit for eliciting distinct or domain-specific homeostatic perturbations [136]. Finally, running pace can be affected by varying wind and temperature conditions, the rigors of training, “the mysteries” of the body and day-to-day variation in recovery and readiness to train. Athletes must therefore cultivate an ability to “feel” the proper intensity, as intensity integrates three forms of feedback: running pace, physiological responses and perception of effort [55]. Intensity scales are imperfect tools, but the above-mentioned potential sources of error seem to be outweighed by the improved communication between coach and athlete that a common scale facilitates [123]. The intensity scale outlined here (Table 4) can be used as a framework for both scientists and practitioners involved in middle-distance running. Still, future training studies should aim to verify whether different methods to prescribe training will affect resulting training execution and adaptation.

Studies of endurance athletes have employed several methods of intensity distribution quantification. These are either anchored around different running paces, standardized blood lactate ranges, “time-in-zone” heart rate analysis based on quantification of the training time spent within different heart rate ranges identified from preliminary threshold testing, or the “session goal” approach where each training session is nominally allocated to an intensity zone based on the intensity of the primary part of the workout [62, 122–124]. Based on the nature and characteristics of available best practice training information [19–59], the session goal approach was used in this review to quantify the intensity distribution for the analyzed running sessions.

### Training Volume

Most world-leading middle-distance runners train about 500–600 h per year, although some 800-m runners may train for less than 400 h [25, 28, 30, 47, 54, 59]. This training volume is 40–70% of what has been reported for successful endurance athletes in cross-country skiing, biathlon, cycling, triathlon, swimming and rowing [110, 112, 127, 128, 137–143]. This difference is likely explained by the fact that running is a weight-bearing locomotion modality where large muscle groups in the lower limbs perform plyometric actions to overcome the vertical and horizontal ground reaction forces involved [99, 144]. The lower amount of training hours in middle-distance runners than the abovementioned sports is mainly due to shorter training sessions with higher degree of neuromuscular loading, and not lower training frequency. Both 800- and 1500-m runners perform approximately 500 training sessions per year [25, 28, 30, 54, 59], similar to other elite endurance athletes [62, 111, 112, 127, 128]. After the competitive season, the training volume is substantially decreased in the transition period when mostly alternative activities and easy runs are performed. Thereafter, the training volume increases gradually, reaching a maximum in the mid-to-late preparation phase, decreasing again as the competition period approaches. The 30–40% reduction in training hours from late-preparation to competition period is in accordance with world-leading athletes in endurance sports such as orienteering, cross-country skiing and biathlon [111, 112, 127, 128]. However, while most of this reduction is related to a decrease/removal of cross-training in these sports, middle-distance runners reduce the amount of low-intensity running and strength/power/plyometric training.

Table 5 shows weekly training volume across season periods for world-class middle-distance runners. While 800-m runners typically cover 50–120 km·week^−1^, 1500-m runners cover 120–170 km·week^−1^ during the mid-to-late preparation period [10, 16, 17, 22–26, 28–32, 34, 36–41, 43–46, 49–51, 54, 59, 133]. The difference is explained by fewer running kilometers for each session for 800-m athletes, as the rate of training sessions are equal for both disciplines. More specifically, typical “long-run” sessions for 800- and 1500-m runners are in the range of 5–10 and 13–17 km, respectively. Although the best practice coaching literature is limited for female athletes, it is reasonable to assume that the ~ 11% slower running velocity in women is compensated for by less covered distance to ensure the same running duration as for the men. In long-distance running, men and women seem to apply the same training duration [62–65]. Table 5 should therefore be interpreted accordingly.

**Table 5:** Weekly training volume for world-class middle-distance runners across the annual cycle

Variable	Early preparation		Mid-to-late preparation		Pre-competition		Mid-competition	
	800 m	1500 m	800 m	1500 m	800 m	1500 m	800 m	1500 m
Weekly training duration (h)^a^	8–13	9–13	9–15	10–15	9–14	9–14	8–13	8–13
Weekly training sessions (*n*)^a^	6–11	8–12	9–12	10–13	8–11	9–12	7–10	8–11
Weekly running volume (km)	40–80	70–120	70–120	120–170	60–100	100–150	50–80	80–140
Weekly running sessions (*n*)	4–7	8–12	6–10	10–13	6–10	10–12	6–9	10–12
Weekly LIT sessions (*n*)	3–6	6–9	3–5	8–11	3–5	7–10	2–5	4–8
Weekly MIT sessions (*n*)^b^	1–2	1–2	1–2	1–2	0–1	1–2	0–1	1–2
Weekly HIT sessions (*n*)^b^	1–3	0–2	1–3	1–3	0–2	1–3	0–2	1–3
Weekly VHIT sessions (*n*)^b^	0–1	n/a	1–2	0–2	1–3	0–2	1–3	1–3

Short-sprint training (SST) is not included in this analysis, as this is rarely the main goal for an entire session in middle-distance runners. The numbers are based on scientific [74, 93] and best practice [2–42] literature

*LIT* low-intensity training, *MIT* moderate intensity training, *HIT* high-intensity training, *VHIT* very high-intensity training

^a^Supplementary training (strength, power, plyometric training and stretching) included

^b^2–4 weekly sessions in total for MIT, HIT and VHIT

Warm-ups and cool downs in conjunction with interval training and strength/power/plyometric sessions make up a large proportion of the total running volume for 800-m runners, while more training sessions for 1500-m athletes are centered around long runs at low to moderate intensity. Interestingly, the difference in running volume between 800- and 1500-m runners is larger than the difference between 1500- and long-distance/marathon runners. World-leading 5–10 km athletes run 120–200 km·week^−1^ [10, 62–64], while top-class marathon runners cover 150–250 km·week^−1^ [62–64]. Based on these running volume distinctions, one could argue that 1500-m runners in general are more long-distance than middle-distance athletes, although high finishing speed is required in slow races [80].

Running accounts for more than 90% of training hours in 1500-m runners, while the remaining training is typically spent on strength/power (core stability, circuits or light weights), drills, plyometrics and stretching [23, 24, 28, 31, 39, 43–45, 49, 64]. Fewer training sessions (70–80%) are dominated by running in 800-m runners, as they perform a greater amount of strength, power and plyometric training [26, 30–32, 36–38, 40, 50, 51].

### Intensity Distribution

Previous studies have shown that elite endurance athletes seem to converge on a typical intensity distribution in which ~ 80% of annual training sessions are dominated by low-intensive work (< 2 mmol·L^−1^ blood lactate) and ~ 20% are dominated by training at or above the anaerobic threshold (e.g., interval training) [9, 17, 123, 124]. While this intensity distribution for running sessions also seems to apply for world-leading 1500-m athletes [23, 24, 28, 31, 39, 43–45, 49, 64], corresponding 800-m runners seem to follow a 70/30- or 60/40-distribution [26, 30–32, 36–38, 40, 50, 51]. However, although 800-m runners perform intensive training sessions more frequently, total effective interval time/distance remain relatively short due to the high intensities with long recovery times between intervals. Hence, approximately 90% of all running sessions for 800-m athletes is performed at low intensity based on the time-in-zone approach, in line with endurance sports [111, 112, 123].

Overall, 1500-m runners perform longer and more frequent training sessions in zone 1 and 2 (based on our 9-zone scale) than 800-m runners throughout the training year [10, 16, 17, 22–26, 28–32, 34, 36–41, 43–46, 48–51, 54, 59]. Substantial differences are also present for the more intensive training sessions. More specifically, 1500-m runners typically follow a pyramidal intensity distribution, while the training pattern in 800-m runners is more clearly polarized. Both groups perform 2–4 weekly intensive training sessions during the preparation phase. These are typically executed in zone 3–5 for 1500-m runners, with a trend towards more zone-3 training (in the form of progressive long runs, anaerobic threshold runs or interval sessions approximately twice a week) over the last 3–4 decades. The intensive training sessions for 800-m runners during the preparation phase are more evenly distributed across zone 3–6.

The differences in the intensive training sessions between 800- and 1500-m runners become even more pronounced when approaching the competition period. During the late-preparation and early-competition period, 800-m runners typically perform 3–4 weekly intensive sessions in zone 3–7 [26, 30–32, 36–38, 40, 50, 51]. Zone-6 intervals are prioritized at the beginning of this period (1–2 weekly sessions), and then replaced with training in zone 7. Indeed, lactate tolerance and lactate production training are characteristic features for middle-distance athletes (800-m runners in particular), as such training rarely occurs among world-leading sprinters [60] or long-distance runners [61–65]. In contrast, 1500-m runners maintain their zone-3 training with 1–2 weekly sessions during the late-preparation and early-competition period [23, 24, 28, 31, 39, 43–45, 48, 49, 64]. Moreover, preparation-phase training for 1500-m runners in zone 4 and 5 is replaced with 1–2 weekly lactate tolerance training sessions (zone 6) in the late-preparation and early-competition period [23, 24, 28, 31, 39, 43–45, 48, 49].

Middle-distance runners perform short-sprint training (SST; zone 8–9) regularly during the annual cycle, but 800-m runners perform SST to a larger degree than 1500-m runners [22–27, 29–32, 34–54, 57–59]. SST is considered a supplement rather than the main goal of separate training sessions and is typically performed during the last part of the warm-up or after easy long runs. It is generally assumed that sprint training should be performed without accumulation of lactic acid [19–21, 52, 54, 57, 59]. Hence, the distances are most commonly in the range of 60–120 m (zone 8), sometimes even shorter (30–60 m; zone 9), and the time/rest between each repetition is sufficient to ensure full recovery. The sprints are typically performed as strides, progressive runs or flying sprints, where the peak rate of acceleration is reduced to minimize lactate accumulation. The technical aspect of running is also highlighted during SST sessions [37, 41]. A widespread notion among coaches is that MSS is inborn and resistant to training adaptation [19–21, 52, 54, 57, 59], and SST is therefore performed to minimize the downsides of aerobic conditioning on MSS. However, studies have shown that well-trained middle-distance runners can improve MSS [145, 146]. According to best practice literature within sprint training, an intensity of ≥ 90–95% of MSS is required to effectively stimulate adaptation [60].

In summary, world-class 800- and 1500-m runners organize their training quite differently, but with no apparent sex differences in intensity distribution within the disciplines. Table 6 shows case study examples of typical training weeks across the annual cycle for an Olympic 800-m champion and a European 1500-m champion. We argue that the training of these two athletes reveals the main distinctions between typical 800- and 1500-m specialists.

**Table 6:** Case study examples of training weeks for an Olympic 800-m champion and European 1500-m champion across the annual cycle

Day	800-m champion (Vebjørn Rodal)	1500-m champion (Arturo Casado)
*Mid-to-late preparation period*		
Mon	M: 8 km long run (z1–2) + 20 min drills E: 3–4 km warm-up (z1–2) + stretching + 10 min drills + 5 × 100 m strides (z8) + 8 × 1000 m *V*O_2max_ intervals (z4), rec. 1 min + 1–2 km cool down (z1) + 4 × 100 m strides (z8)	M: 14 km long run (z1) E: 10 km long run (z1)
Tue	M: Rest E: Warm-up with basketball + stretching + 10 min drills + 5 × 100 m strides (z8) + 3 × (3 × 4) vertical & (3 × 5) horizontal jumps + 20 × 20–75 steps of running and jumping in stairs with walk down rec. and 6 min set-break + core exercises 20 min	M: 14 km long run (z1) + Drills + Hurdles technique + 10 × 400 m lactate tolerance training (z6), rec. 1 min + 1 km cool down (z1) E: 10 km long run (z1)
Wed	M: 8 km long run (z1–2) + 5 × 100 m strides (z8) + stretching E: 4 km warm-up (z1–2) + 10 min drills + 5 × 80 m strides (z8) + 2 × 10 × 200m lactate tolerance training (z6), rec. 1 min and set-break 6 min + 1–2 km cool down (z1) + stretching	M: 3 km warm-up (z1) + Strength training + 14 km long run (z1) + 18 × 100 m hill repeats (z7/8), rec. easy jog back + 3 km cool down (z1) + plyometrics E: Rest
Thu	M: 3 km warm-up (z1–2) + plyometrics and strength training without weights 2 × 10 exercises (20/20 s work/recovery) E: 4 km warm-up (z1–2) + stretching + 10 min drills + 5 × 100 m strides (z8) + 10 × 1 min *V*O_2max_ intervals (z5), rec. 1 min + 1 km cool down (z1) + 4 × 100 m strides (z8)	M: 4 km warm-up (z1) + 10 × 1000 m *V*O_2max_ intervals (z4), rec. 1:30 min + 3 km cool down (z1) E: 10 km long run (z1)
Fri	M: Rest E: 6 km warm-up (z1–2) + stretching + 10 min drills + 5 × 100 m strides (z8) + 3 × 200 m lactate production training (z7) and 3 × 100 m sprint (z8), rec. 4 min and set-break 8 min + 1 km (z1)	M: 3 km warm-up (z1) + Strength training + 16 km long run (z1/2) + drills + 6 × 100 m strides (z8) E: Rest
Sat	M: 3 km warm-up (z1–2) + stretching + 10 min drills + 5 × 100 m strides (z8) + 90 min. explosive weight training E: 12 km progressive run (z1–3)	M: 4 km warm-up (z1) + 2 × 6000 m anaerobic threshold intervals (z3), rec. 2 min + 3 km cool down (z1) E: Rest
Sun	M: 2 h long run (z1) E: Rest	M: 12 km long run (z1) E: Rest
	*Weekly total of* ~ *110 km (75% LIT, 17% HIT, 4% VHIT and 4% SST)*	*Weekly total of* ~ *152 km (81% LIT, 8% MIT, 7% HIT, 3% VHIT and 1% SST)*
Mon	M: 6 km warm-up (z1–2) + 20 min. drills E: 4 km warm-up (z1–2) + 10 min. drills + 5 × 100 m strides (z8) + 2 × 10 × 200m lactate tolerance training (z6), rec. 1 min and set-break 6 min + 1–2 km cool down (z1)	M: 14 km long run (z1) + Drills + Hurdles technique + 15 × 200 m lactate tolerance training (z6) with 100 m easy jog in between (each 200 m in 29 s on average + 1 km cool down (z1) E: 10 km long run (z1)
Tue	M: 12 km long run (z1) E: Warm-up with basketball + stretching + 10 min drills + 5 × 100 m strides (z8) + 3 × (3 × 4) vertical and (3 × 5) horizontal jumps + 15 × 20–75 steps of running and jumping in stairs with walk down rec. and 6 min. set-break + 20 min. core exercises	M: 3 km warm-up (z1) + Strength training + 5 km (z1) + Fartlek (5, 4, 3, 2, 3 and 2 min. running in z3 with easy jog z1–2 in between corresponding to half the repetition time) + 1 km cool down (z1) E: 10 km long run (z1)
*Pre-competition period*		
Wed	M: 6 km warm-up (z1–2) + 4 × 100 m strides (z8) + stretching E: 4 km warm-up (z1–2) + 10 min drills + 6 × 100 m strides (z8) + 4 × 300 m and 4 × 100 m lactate production training (z7), rec. 4 min and set-break 8 min + 1 km cool down (z1) + stretching	M: 14 km long run (z1) + 12 × 100 m hill repeats (z7/8), rec. easy jog back + 3 km cool down (z1) + plyometrics E: Rest
Thu	M: 5 km warm-up (z1) + 20 min drills E: 4 km warm-up (z1–2) + 10 min drills + 5 × 100 m strides (z8) + 10 × 1 min *V*O_2max_ intervals on treadmill (z5), rec. 1 min + 1–2 km cool down (z1)	M: 6 km warm-up (z1) + drills + 5 × 1200 m *V*O_2max_ intervals (z5), rec. 3 min + 1 km cool down (z1) E: 10 km long run (z1)
Fri	M: Rest E: 3 km warm-up (z1–2) + stretching + 10 min drills + 5 × 100 m strides (z8) + 3 × (150–120–100 m) lactate production training (z7), rec. 3 min and set-break 6 min + 1–2 km cool down (z1)	M: 3 km warm-up (z1) + Strength training + 16 km long run (z1) + drills + 3 × 3 × 60 m sprints (z8), rec. walk back and set-break 3 min E: Rest
Sat	M: 3 km warm-up (z1–2) + plyometrics and strength training without weights 2 × 10 exercises (20/20 s work/recovery) E: 4 km warm-up (z1–2) + 3 × 600 m, 3 × 400 m and 3 × 200 m lactate tolerance training (z6), rec. 4 min and set-break 10 min + 1 km cool down (z1) + stretching	M: 4 km warm-up (z1) + 8 km AT run (z3) + 4 km cool down (z1) E: Rest
Sun	M: 15–20 km long run (z1) on road or treadmill E: Rest	M: 14 km long run (z1) E: Rest
	*Weekly total of* ~ *100 km (77% LIT, 10% HIT, 10% VHIT and 3% SST)*	*Weekly total of* ~ *147 km (82% LIT, 11% MIT, 4% HIT, 2% VHIT and 1% SST)*
*Competition period*		
Mon	M: 5 km warm-up (z1–2) + 4 × 100 m strides (z8) + stretching E: 3 km warm-up (z1–2) + 10 min drills + 5 × 100 m strides (z8) + 200–400–600–600–400–200 m lactate tolerance training (z6) rec. 1–3 min + 1 km cool down (z1) + stretching	M: 6 km warm-up (z1) + drills + 3 × 4 × 200 m lactate tolerance training (at 25 s on average; z6), rec 1 min and set-break 3 min + 1 km cool down (z1) E: 6 km recovery run (z1)
Tue	M: Rest E: 6 km warm-up (z1–2) + 10 min drills + 4 × 4 × 4 vertical and 6 × 30 m horizonal jumps, rec. 1 min + 10 × 20–30 jumps in stairs with walk down rec	M: 6 km AT run at 3:10 min. per km (z3) + 6 km fartlek [4 × (1 km in 3 min and 500 m in 1:40 min), 18:40 in total (z3)] + 2 km (z1) E: Rest
Wed	M: 8 km long run (z1–2) + 4 × 100 m strides (z8) E: 3 km warm-up (z1–2) + 10 min drills + 5 × 100 m strides (z8) + 2 × 5 × 200 m lactate tolerance/production training (z6–7), rec. 2 min and set-break 10 min + 1 km cool down (z1)	M: 6 km warm-up (z1) + drills + 4 × 1000 m *V*O_2max_ intervals (2:30 min. on average, z5), rec. 3 min. + 2 km cool down (z1) E: 6 km recovery run (z1)
Thu	M: Rest E: 3 km warm-up (z1) + 10 min drills + 5 × 100 m strides (z8) + 5 × 100 m near-maximal sprints (z8), 1–2 min rec. + plyometrics and strength training without weights 20 min	M: 12 km long run (z1) + Strength training + drills + 6 × 100 m strides (z8) E: Rest
Fri	M: Rest E: 3 km warm-up (z1–2) + 10 min drills + 4 × 100 m strides + 2 × 400 m lactate production training (z7), rec. 10 min + 1–2 km cool down (z1)	M: 6 km warm-up (z1) + drills + 3 × 4 × 300 m lactate tolerance training, each run at 40 s on average (z6), rec. 1 and set-break 3 min + 1 km cool down (z1) E: 6 km recovery run (z1)
Sat	M: 5 km warm-up + 10 min drills + 4 × 100 m strides (z8) E: 4 km warm-up (z1–3) + drills, strides and speed work + 800 m competition + 1–2 km cool down (z1)	M: 15 km long run (z1) + drills + 6 × 100 m strides (z8) E: Rest
Sun	M: 8 km recovery run (z1) E: Rest	M: 6 km warm up (z1) + drills + 8 × 150 m lactate production training with 4 kg ballast (at 16–17 s on average; z7), rec. 3 min. + 1 km cool down (z1) E: 6 km recovery run (z1)
	*Weekly total of* ~ *63 km (84% LIT, 10% VHIT and 6% SST)*	*Weekly total of* ~ *106 km (77% LIT, 11% MIT, 4% HIT, 7% VHIT and 1% SST)*

Zone 1 running was performed at ~ 3:40 min·km^−1^ for Casado and ~ 3:55 min·km^−1^ for Rodal. Drills for Rodal consisted of 2–3 × 30 m ankle triplings, high knees, butt kicks, straight-leg bounce and other running-specific exercises. Drills for Casado consisted of 2 × 40 m high knees, butt kicks, straight-leg bounce, forward skip, double leg bounce, forward lung and brief sprinting. Hurdle technique for Casado consisted of four exercises over 6–8 hurdles with 1–2 steps in between. Strength training for Rodal consisted of explosive full and half squats, step-ups for each leg, bench flies, arm-movement simulations and toe lift at 30–70% of one repetition maximum, in addition to running-specific core exercises without weights. Strength training for Casado consisted of 3 × (6 × full squat, dead lift, step-ups for each leg, bench press at 50–70% of one repetition maximum, plus other exercises such as hamstring curls, calf raises and core strength. Stairwork for Rodal consisted of high-frequency running, one-leg and two-leg jumps. Plyometrics for Casado consisted of 2 × 50 m one-leg bounding for each leg and 2 × 50 m two-leg bounding on grass. Vertical plymetrics for Rodal consisted of hurdle jumps, while horizontal plyometrics consisted of one-leg jumps and various steps/”sprunglauf” exercises. The training program for Casado was designed by Arturo Martín-Tagarro

*M* morning session, *E* evening session, *z *training zone (see Table 4), *AT *anaerobic threshold

### Strength, Power and Plyometric Training

A review of the best practice literature reveals that most world-class middle-distance runners perform regular strength, power and plyometric training as a supplement to their specific running conditioning [22–59]. This training is typically executed as a combination of (1) core strength/stability (static or dynamic sit-ups and back exercises), (2) strength training with machines or free weights (e.g., half squats, cleans, lunges, step ups, leg press, leg curl, leg extension) without causing significant hypertrophy, (3) circuit training with body mass resistance, (4) medicine ball exercises, and (5) vertical and horizontal multi-jumps on grass, inclines, stairs (e.g., bounding, skipping, squat jumps, hobbling, springing) or jumping over hurdles. Combinations of running and circuit training exercises have also been applied (e.g., 8–10 exercises with 1 K running in between) [36, 53]. In general, the supplementary training is poorly described in terms of resistance loading, sets and repetitions, and caution must therefore be exercised when drawing conclusions. However, two main features become apparent after reading the best practice literature: more supplementary training is performed during the preparation (typically 2–4 times per week) than competition (0–2 times per week) period, with 800-m runners of both sexes performing such training more frequently than corresponding 1500-m runners. Future studies should aim to concretize more detailed recommendations for middle-distance runners regarding types of exercises, resistance loading, sets and repetitions.

Based on experimental evidence, adding supplementary training on 2–3 occasions per week in the form of strength, power and plyometric training appears to improve running economy, time trial performance and MSS in middle- and long-distance runners across a broad performance range [4, 147–149]. In contrast, a causal relationship between core stability, athletic performance and injury risk has not been established [150].

## Tapering

While the training components across the annual cycle of world-class middle-distance runners are described considerably more in detail in best practice versus research literature, tapering represents an area where more information can be obtained from scientific studies. Although potential differences in tapering strategies between 800- and 1500-m runners cannot be identified based on current available information, it is reasonable to assume that the training volume is lower and the key workouts are shorter and more intensive for 800-m runners during this period.

The general scientific guidelines for a likely effective taper in endurance-related sports are a 2- to 3-week period incorporating 40–60% reduction in training volume following a progressive non-linear format, while training intensity and frequency are maintained or only slightly reduced [151–155]. However, although individual differences are clearly present, tapering length increases with competition distance, and approximately 1 week seems sufficient for middle-distance athletes [33, 56, 156, 157]. Spilsbury and associates reported that elite middle-distance runners perform three interval training sessions on average during the last tapering week [157]. Each of these interval sessions are typically executed at race pace with a total distance of ~ 2 K. This corresponds to ~ 50% of the total interval distance in the preceding weeks of the tapering period. It should be noted that a sub-group bias may have affected the outcomes in this study, as the British middle-distance sample included twice the number of 1500-m runners (*n* = 12) than 800-m runners (*n* = 6).

According to studies of well-trained endurance athletes, a realistic performance goal for the final taper should be a competition performance improvement of about 2–3%, corresponding to 2–4 and 4–6 s in world-leading 800- and 1500-m runners, and this is due to positive changes in the cardiorespiratory, metabolic, hematological, hormonal, neuromuscular and psychological status of the athletes [151–155]. However, based on annual performance changes in world-leading middle-distance contestants [87], we argue that the performance gains suggested in research literature are likely smaller for athletes of higher standards.

## Conclusions

This review integrated scientific and best practice coaching literature regarding the training and development of elite middle-distance performance. To this end, we have outlined a framework for specific characteristics (e.g., training methods, volume and intensity) and identified the training differences between 800- and 1500-m runners in detail. Overall, the training of 800-m athletes consists of considerably lower running volume, a higher proportion of interval training at or above the anerobic threshold and more supplementary work in form of strength, power and plyometric training compared to 1500-m runners. These features seem to reflect the divide in physiological demands separating these two middle-distance disciplines. Although there are many studies focusing on middle-distance running, there is a considerable gap between science and best practice in how training principles and methods are applied, highlighting the need for future investigations employing a more holistic approach. For example, training differences and assessment of mechanical and physiological capacities of elite middle-distance runners throughout the training year and over several seasons should be observed. Such approaches would establish mechanistic connections between training content, changes in performance and underlying mechanical and physiological determinants. The conclusions drawn in this review may serve as a position statement and provide a point of departure for forthcoming studies regarding training and development of elite middle-distance runners.

## Declarations

### Availability of data and materials

All data and materials support the published claims and comply with field standards.
